# Effect of energy source and level, animal age, and sex on the flavor profile of sheep meat^1,2^

**DOI:** 10.1093/tas/txaa081

**Published:** 2020-06-11

**Authors:** Jerad R Jaborek, Henry N Zerby, Macdonald P Wick, Francis L Fluharty, Steven J Moeller

**Affiliations:** 1 Department of Animal Sciences, The Ohio State University, Columbus, OH; 2 Department of Animal Sciences, The Ohio State University, Wooster, OH

**Keywords:** age, energy source, lamb flavor, off-flavor, sex

## Abstract

The effects of dietary energy source, energy level, sheep age, and sheep sex on flavor and off-flavor intensity were evaluated. Consumer panelists, with previous lamb-eating experience, assessed lamb flavor and off-flavor intensity on a 100-point, end-anchored scale (0 = very mild to 100 = very intense), with off-flavor being defined as anything different than lamb flavor. Lamb longissimus thoracis (LT) and whole, boneless ground shoulder (GS) formed into patties were evaluated. Trial 1 was a randomized complete block design with a 3 × 2 factorial arrangement of treatments. Sheep age (ewe lambs, yearling ewes, and mature ewes; *n* = 16/age) and ad libitum access to diets [alfalfa pellets (AP) or whole-shelled corn (WSC100)] were treatments. The LT from mature ewes had a greater (*P* ≤ 0.02) off-flavor intensity when compared with yearling ewes and ewe lambs. Ground shoulder from sheep raised on AP had a greater lamb flavor (*P* ≤ 0.04) and off-flavor (*P* ≤ 0.04) intensity than GS from sheep consuming WSC100. Trial 2 was a randomized complete block design with a 3 × 2 × 2 factorial arrangement of treatments. Three dietary treatments [AP, WSC100, and restricted intake of whole-shelled corn to 85% of ad libitum (WSC85)], lamb sex (ewes and wethers; *n* = 48/sex), and lamb age [short fed, 177 ± 16.6 d of age and 93 ± 20.5 d on feed (DOF); long fed, 294 ± 7.0 d of age and 219 ± 3.8 DOF]. Flavor intensity of the LT was greater (*P* ≤ 0.05) from lambs offered AP when compared with lambs offered WSC85, whereas lamb flavor of the LT from lambs fed WSC100 was intermediate and not different from the lamb flavor of the LT of lambs fed AP or WSC85. The LT of long-fed lambs had a greater (*P* ≤ 0.01) lamb flavor and off-flavor intensity when compared with short-fed lambs. Lambs offered AP resulted in a GS with greater lamb flavor intensity (*P* ≤ 0.01) when compared with lambs offered WSC85 and WSC100, with no diet influence on GS off-flavor intensity. Long-fed lambs produced GS with a greater lamb flavor (*P* ≤ 0.01) and off-flavor (*P* ≤ 0.01) intensity when compared with GS from short-fed lambs. Results from the two trials indicate lamb flavor and off-flavor intensity were greater from sheep offered a high-forage (AP) diet when compared with a high-concentrate (WSC) diet. Lamb flavor intensity increased as age of the sheep at harvest increased, suggesting dietary management and associated age-related effects at harvest will influence consumer perception of lamb flavor.

## INTRODUCTION

The American Lamb Resource Center (2013) findings indicated that the American sheep industry should focus on meat product characteristics in order to improve the consumer’s eating experience, with a goal of providing consumers with a consistent and premier lamb product every time. The 2015 National Lamb Quality Audit (Hoffman et al., 2016) identified eating satisfaction as the most important quality attribute for consumers when making a decision to purchase lamb, with eating satisfaction and quality most commonly described as either lamb flavor or taste by respondents. Although few differences in meat quality characteristics, including pH, instrumental color, cook loss, and shear force, were reported due to diet, sheep age, and sex (Jaborek et al., 2018a), the influence of these classifications on lamb flavor has been reported to be variable, which can present inconsistent eating experiences for consumers. Results from the National Lamb Quality Audit (Hoffman et al., 2016) indicated that the “inconsistency of lamb flavor” was a threat to lamb consumption in the United States. Consumer acceptance of and preference for lamb flavor characteristics have been reported to vary across geographical locations (Prescott et al., 2001; Font i Furnols et al., 2006), compounding the challenge of identifying and consistently providing an ideal lamb flavor profile.

The present study investigates the effects of dietary energy source and level, sheep age, duration of feeding period, and sheep sex on lamb flavor intensity and off-flavor intensity of sheep meat as determined by a consumer panel. The authors hypothesized that offering a legume-based diet would increase the lamb flavor intensity of sheep meat when compared with meat from sheep offered a concentrate-based diet. In addition, lamb flavor intensity and off-flavor intensity were hypothesized to increase in meat from older sheep when compared with meat from younger lambs.

## MATERIALS AND METHODS

### Sheep Rearing and Treatments

Animal procedures were approved by the Institutional Animal Care and Use Committee (IACUC #2015A00000023) of The Ohio State University. The experiments were conducted at the Ohio Agriculture Research and Development Center (Wooster, OH) sheep research feedlot inside a covered barn. Sheep were housed on slatted floor pens with fence line feeding bunks and provided ad libitum access to water.

Forty-eight traditional market weight and age lambs, 48 long-fed lambs, 16 yearling ewe lambs, and 16 mature ewes all of Dorset × Hampshire breed composition were used in two experimental trials. Trial 1 utilized ewe lambs [174 ± 20.0 d of age (DOA)], yearling ewes 421 ± 11.6 DOA, and mature ewes (>1,095 DOA; *n* = 16/age group) offered ad libitum access to either whole-shelled corn (WSC100) or alfalfa pellets (AP). In trial 2, ewe and wether lambs (*n* = 48 per sex) were offered one of three dietary treatments [ad libitum access to AP or WSC100 or whole-shelled corn at 85% of ad libitum intake (WSC85)] for either 93 ± 20.5 d (short‐fed lambs; 177 ± 16.6 DOA) or 219 ± 3.8 d (long‐fed lambs were 294 ± 7.0 DOA). Sheep were blocked by sex and age, stratified by initial body weight (BW), assigned to 32 pens with four sheep per pen, and randomly assigned a dietary treatment.

### Performance and Carcass Data Collection

Feeding methods and performance data collection procedures were presented previously for short-fed lambs (Jaborek et al., 2017), long-fed lambs (Jaborek et al., 2018b), and yearling and mature ewes (Jaborek et al., 2018a). Short-fed lambs from Jaborek et al. (2017) were removed from the feedlot for harvest on a pen basis when ewe and wether lambs reached an average BW of 59.0 and 63.5 kg, respectively. Long-fed lambs from Jaborek et al. (2018b) were removed from the feedlot for harvest on a pen basis with a similar number of DOF for each of the three dietary treatments. Yearling and mature ewes were removed from the feedlot for harvest after being fed for an average of 63.5 and 71.5 d, respectively. All sheep were harvested, and carcasses were fabricated, at The Ohio State University Meat Science Laboratory (Columbus, OH). Carcass data collection and meat sample collection procedures were reported by Jaborek et al. (2017, 2018a, 2018b). Longissimus thoracis (LT) were removed at fabrication, vacuum packaged, and aged for a total of 14 d prior to being frozen (−20 °C) and stored until taste panel evaluation. A boneless, square cut shoulder from each carcass was vacuum packaged and aged for a total of 14 d, then ground once through a 0.95-cm plate, packaged, and frozen (−20 °C). Muscle samples were thawed overnight at 3 °C and the ground shoulder (GS) was formed into patties prior to cooking.

### Taste Panel Procedure

The LT and the GS patty were grilled on a clam style grill set at 190 °C. The LT was cooked to 65 °C internal temperature to replicate a degree of doneness more commonly used among the general population, whereas the GS patties were cooked to 71 °C to ensure food product safety. Sensory samples were cut to form 1.5-cm cubes. Cubed samples were held at 65 °C until consumption in either a 50-mL centrifuge tube (sample days 1 and 2) or in disposable Ziploc bags (sample days 3 and beyond). The switch to disposable bags resulted from the detection of an aroma in cleaned conical tubes on day 2 of consumer testing.

A consumer panel was used in lieu of a trained sensory panel. Consumer responses were sought to address the findings and industry-suggested approaches to understanding lamb flavor per the 2015 National Lamb Quality Audit (Hoffman et al., 2016). Consumer level perceptions of flavor and off-flavor intensity from persons known to eat lamb and not be averse to lamb flavor or lamb flavor variation were used in the present study. The consumer taste panel was comprised of graduate students, staff, and faculty from The Ohio State University, Department of Animal Sciences (Columbus, OH). Taste panelists had prior experiences eating lamb and were identified as not being aversive to lamb.

Taste panelists were asked not to eat 1 h before each tasting session to limit any taste conflicts from previous meals. Taste panels were conducted three times each test day at 0900, 1200, and 1500 hours, offering each panelist eight unique samples per session. Samples provided to the taste panel were blocked by diet, animal age, and sex and served in a random order within each taste panel session. Panelists were instructed to chew the sample for 5 to 10 s to experience the flavor profile of the meat sample and asked to discard rather than consume the samples following assessment. Between samples, panelists were provided with unsalted crackers, apple juice, and distilled water to cleanse their palate.

Panelists were asked to rate lamb flavor intensity on an end-anchored line scale from 0 to 100, with 0 being very mild and 100 being very intense. Off-flavor intensity was rated by consumer panelists on an end-anchored line scale from 0 to 100, with 0 being very mild and 100 being very intense. Specific off-flavors were recorded by panelist when detected, with off-flavors being defined as anything other than lamb/sheep meat flavor. Specific off-flavors options offered to panelists on the sample ballots included sweet, sour, salty, bitter, umami/meaty, browned, metallic, livery, bloody, grassy, fecal/barnyard, urine/ammonia, and an option to write in another off-flavor descriptor(s). Unfortunately, consumers were not specifically asked if they liked the flavor or off-flavor intensity in the present study.

### Statistical Analysis

The experimental design for trial 1 was a randomized complete block design with a 3 × 2 factorial arrangement of treatments to distinguish differences between diet (AP and WSC100) and sheep age (lamb, yearling, and mature). Statistical analyses was performed using the GLIMMIX procedure in SAS (SAS Inst. Inc., Cary, NC) with a lognormal distribution to account for lack of normality (positive skewness). The statistical model used was: Yijklm = μ + Di + Aj + DAij + pk + vl + eijklm, where Di = diet fed, Aj = sheep age, and DAij = the interaction between the diet and sheep age, all as fixed effects, and pk = pen (nested within diet by age) and vl = panelist as the random effects, and eijklm = random residual error.

The experimental design for trial 2 was a randomized complete block design with a 3 × 2 × 2 factorial arrangement of treatments to distinguish differences between diet (AP, WSC100, and WSC85), sheep age (short-fed lambs and long-fed lambs), and sex (wether and ewe). Statistical analysis was performed using the GLIMMIX procedure in SAS (SAS Inst. Inc., Cary, NC) with a lognormal distribution. The statistical model used was: Yijklmn = μ + Di + Aj + Sk + DAij + DSik + SAjk + DASijk + pl + vm + eijklmn, where Di = diet, Aj = sheep age, and Sk = the sex of the lamb, DAij = the interaction between the diet and age, DSik = the interaction between the diet and sex, SAjk = the interaction between the age and sex, and DASijk = the interaction between the diet, age, and sex, all as fixed effects, and pl = pen (nested within diet by age) and vm = panelist, as the random effects, and eijklmn = random residual error.

The frequency of reported observations of the individual off-flavor categories were analyzed using the GLIMMIX procedure in SAS (SAS Inst. Inc., Cary, NC) with a binary distribution in accordance with each of the aforementioned models. If the off-flavor was not detected, it was set = 0; if the off-flavor was detected, it was set = 1.

The LSMEANS and DIFF statements were used to obtain estimates of treatment means and SEs and to distinguish differences between treatment levels. At *P* ≤ 0.05, differences amongst treatments were considered significant. Tendencies were identified as 0.05 < *P* ≤ 0.10. Data were back-transformed to the 0 to 100 end-anchored scale for interpretation consistent with taste panel scoring procedures.

## RESULTS AND DISCUSSION

### Trial 1

Lamb flavor intensity score (Table 1) from the LT samples was not influenced by dietary treatment (*P* = 0.61; WSC100 vs. AP) or by sheep age classification (*P* = 0.28; lamb, yearling, or mature ewe). Off-flavor intensity score for LT samples was not different between WSC100 and AP diets (*P* = 0.28); however, off-flavor intensity score was greater (*P* ≤ 0.02) for the LT from mature ewes when compared with LT derived from ewe lambs and yearling ewes. Of note, consumer panelists tended (*P* = 0.10) to detect a livery flavor more frequently in LT samples from sheep offered AP when compared with sheep offered WSC100 (numerical results not presented). The umami/meaty flavor tended (*P* = 0.10) to increase in LT samples as sheep age increased (numerical results not presented).

**Table 1.** Flavor intensity of cooked LT and GS patty samples from female sheep of different ages and offered different energy sources

	Diet*			Sheep age			
Item	WSC	AP	SEM†	Lamb	Yearling	Mature	SEM†
Natural log transformation							
LT lamb flavor intensity	3.51	3.55	0.110	3.47	3.51	3.61	0.115
LT off-flavor intensity	0.30	0.57	0.439	0.09^d^	0.12^d^	1.08^c^	0.455
GS lamb flavor intensity	3.46^b^	3.67^a^	0.103	3.45	3.63	3.62	0.110
GS off-flavor intensity	−0.08^b^	0.53^a^	0.506	−0.05	0.29	0.43	0.522
Back transformation‡							
LT lamb flavor intensity	33.55	34.77	—	32.21	33.45	36.97	—
LT off-flavor intensity	1.34	1.76	—	1.10^d^	1.12^d^	2.95^c^	—
GS lamb flavor intensity	31.78^b^	39.19^a^	—	31.37	37.62	37.25	—
GS off-flavor intensity	0.92^b^	1.70^a^	—	0.62	1.26	1.54	—

*WSC = ad libitum access to a whole shelled corn diet and AP = ad libitum access to an alfalfa pellet diet.

†The reported SEM is the greatest between the levels within the treatment.

‡The back transformation is on a 0 to 100 scale, where 0 = very mild and 100 = very intense.

^a, b^Diet means within a row without a common superscript letter differ (*P* ≤ 0.05).

^c,d^Sheep’s age means within a row without a common superscript letter differ (*P* ≤ 0.05).

Ground shoulder patty samples from sheep offered AP resulted in greater lamb flavor (*P* ≤ 0.04) and off-flavor (*P* ≤ 0.04) intensity scores when compared with the GS from sheep offered WSC100 (Table 1). Similarly, Crouse et al. (1981) reported that the flavor of patties, made from the loin with added fat, from lambs offered an AP diet was more pronounced when compared with the flavor of patties from lambs offered a concentrate diet. Consumer panelists tended (*P* = 0.08) to more frequently detect umami/meaty flavors in the GS samples from sheep offered AP when compared with sheep offered WSC100 (numerical results not presented). Lamb flavor (*P* = 0.16) and off-flavor (*P* = 0.28) intensity scores of the GS were not affected by age of sheep at harvest in the present study. In contrast, results of a profile taste panel conducted by Jeremiah et al. (1998) indicated greater lamb flavor intensity in the boneless shoulder from younger lambs (3–9 months of age) when compared with older lambs (9–15 months of age). Jeremiah et al. (1998) also reported a greater off-flavor intensity with increased animal age and the noted presence of “muttony” flavor in product from the oldest lambs. In the present study, consumer panelists tended (*P* = 0.10) to note and report greater levels of fecal/barnyard flavors in the GS samples as the age of sheep at harvest increased (numerical results not presented).

The compounds 4-methyloctanoic acid, 4-methylnonanoic acid, and 4-ethyloctanoic acid within the fat depots of sheep were identified as the primary factors contributing to species-specific flavors observed through volatilization upon cooking (Wong et al., 1975a, 1975b; Brennand et al., 1989). Concentrations of these species-specific flavors deposited in the carcass fat can vary depending on a variety of factors, such as the sheep’s age, sex, and the diet consumed, causing variations in perceived flavor intensity (Sutherland and Ames, 1996; Young et al., 1997; Young et al., 2003; Watkins et al., 2010). Brennand and Lindsay (1992) have demonstrated that the species-specific compounds found in the carcass fat (subcutaneous, perineal, and intramuscular adipose tissue) can also vary by anatomical location (rump, breast, shoulder, and leg) on the sheep carcass. In the present study, consumer panelists were more likely to discern treatment differences in the GS sample compared with the LT sample, a finding very consistent with the greater overall fat content within the GS samples (Jaborek et al., 2018a). In addition, grinding exposes a greater proportion of surface area when compared with a sample derived from a whole muscle product, thereby enhancing the consumer panelists’ ability to identify the volatile compounds and distinguish greater treatment differences. Results from this portion of the study demonstrate a pattern for greater lamb flavor and off-flavor intensity in the meat from sheep offered a high-forage diet (AP) when compared with a high-concentrate diet (WSC100) and greater off-flavor intensity in the meat from older sheep compared with younger sheep.

### Trial 2

Longissimus thoracis samples from lambs offered WSC85, a dietary treatment designed to reduce growth rate and the deposition of fat, had a lesser (*P* ≤ 0.05) lamb flavor intensity than lambs offered WSC100 or AP (Table 2). Results of the present study disagree with the results of Borton et al. (2005), who reported that loin chops from lambs offered a concentrate diet had a greater lamb flavor intensity when compared with loin chops from lambs grazing ryegrass. However, off-flavor intensity score tended (*P* = 0.06) to be greater in LT samples from sheep offered AP, which is in agreement with the results of Borton et al. (2005), who reported fewer off-flavors from lambs offered a concentrate diet when compared with loin chops from lambs grazing ryegrass. The observed trend for increased off-flavor intensity from sheep offered a legume-based (AP) diet is also supported by findings of grazing research comparing legume pastures with grass pastures, whereby lamb flavor and off-flavors were reported to increase from sheep offered legume pasture (Cramer et al., 1967; Shorland et al., 1970). Young et al. (2003) and Schreurs et al. (2007) reported that skatole (3-methylindole) is the compound primarily responsible for pastoral flavors that contributes to “muttony” flavors in sheep meat. A trained sensory panel assessment conducted by Priolo et al. (2002) reported a greater “typical” lamb flavor in loin steaks from lambs offered a concentrate diet when compared with grass-fed lambs. Long-fed lambs had a greater lamb flavor intensity (*P* ≤ 0.01; Table 2), which may correspond with greater intramuscular fat within the LT of long fed lambs (5.12% vs. 3.72%) when compared with short-fed lambs (Jaborek et al., 2018a). Off-flavor intensity was also greater (*P* ≤ 0.01) in the LT when compared with the LT derived from shorter-fed lambs. Likewise, Borton et al. (2005) reported loin chops from heavy weight (77 kg) lambs to have more off-flavors when compared with lighter weight (52 kg) lambs. In contrast, Crouse et al. (1981) reported no differences between the flavor scores of ground LT patties from lambs slaughtered at 62 kg when compared with contemporary lambs that remained in the feedlot for an additional 63 d before being harvested at 77 kg. The observation of no difference in flavor profile by Crouse et al. (1981) may be directly related to total days on feed, which was 126 d in the present study and 63 d reported by Crouse et al. (1981), and the resulting greater end weight and age achieved in the present study. The additional time on feed and weight end point may have combined to allow the diet to further change fatty acid composition of the muscles, thereby changing flavor.

**Table 2.** Flavor intensity of cooked LT and GS patty samples from lambs of different age, sex, and offered different energy sources at different levels

	Diet*				Lamb age			Sex		
Item	WSC100	AP	WSC85	SEM†	Short fed	Long fed	SEM†	Ewe	Wether	SEM†
Natural log transformation										
LT lamb flavor intensity	3.56^ab^	3.59^a^	3.45^b^	0.157	3.44^f^	3.63^e^	0.156	3.56	3.51	0.156
LT off-flavor intensity	0.47	0.71	0.21	0.492	0.21^f^	0.71^e^	0.487	0.43	0.49	0.487
GS lamb flavor intensity	3.49 ^d^	3.67^c^	3.44^d^	0.102	3.45^f^	3.62^e^	0.099	3.57	3.50	0.099
GS off-flavor intensity	0.32	0.15	0.42	0.463	0.01^f^	0.60^e^	0.454	0.47	0.13	0.455
Back transformation‡										
LT lamb flavor intensity	35.16^a^	36.28^a^	31.63^b^	—	31.09^f^	37.83^e^	—	35.05	33.56	—
LT off-flavor intensity	1.60	2.03	1.23	—	1.24^f^	2.04^e^	—	1.54	1.64	—
GS lamb flavor intensity	32.66^d^	39.41^c^	31.17^d^	—	31.49^f^	37.21^e^	—	35.46	33.05	—
GS off-flavor intensity	1.38	1.16	1.52	—	0.99^f^	1.82^e^	—	1.60	1.13	—

*WSC100 = ad libitum access to a whole-shelled corn diet, AP = ad libitum access to an alfalfa pellet diet, and WSC85 = whole-shelled corn diet offered at 85% of ad libitum computed from WSC100 contemporaries.

†The reported SEM is the greatest between the levels within the treatments.

‡The back transformation is on a 0 to 100 scale, where 0 = very mild and 100 = very intense.

^a,b^Diet means within a row without a common superscript letter differ (*P* ≤ 0.05).

^c,d^Age means within a row without a common superscript letter differ (*P* ≤ 0.05).

Off-flavors, specifically livery and fecal/barnyard flavors, tended (*P* = 0.10) to be detected by consumer taste panelists more frequently in LT samples from long-fed lambs when compared with short-fed lambs (results not presented). Sex of the lamb did not affect lamb flavor (*P* = 0.33) and off-flavor (*P* = 0.68) intensity scores of LT samples.

Lamb flavor intensity scores from GS samples were greater (*P* ≤ 0.01) from lambs offered AP when compared with samples from lambs offered WSC (WSC100 or WSC85; Table 2). Off-flavor intensity scores from GS were not affected (*P* = 0.45) by diet. Ground shoulder patty samples from long-fed lambs had a greater lamb flavor intensity (*P* ≤ 0.01) and off-flavor intensity (*P* ≤ 0.01) when compared with short-fed lambs. Consumer taste panelists detected fecal/barnyard off-flavors more frequently (*P* ≤ 0.05) in GS samples derived from long-fed lambs when compared with short-fed lambs (numerical results not presented). Whereas no sex effects (*P* = 0.13) were observed for GS lamb flavor intensity, off-flavor intensity tended (*P* = 0.06) to be greater in GS samples from ewes compared with wether lambs. This finding may be due to a trend for a livery off-flavor being detected (*P* = 0.09) more frequently by consumer taste panelists when GS patty samples were from ewe lambs compared with wether lambs (numerical results not presented). In contrast, Jeremiah et al. (1998) reported that ewe lambs had a greater lamb flavor and a greater overall acceptable flavor when compared with wether lambs. Results from this portion of the study demonstrate a pattern for greater lamb flavor in the meat from sheep offered a high-forage diet (AP) when compared with a high-concentrate diet (WSC85) and greater lamb flavor and off-flavor intensity in the meat from long-fed lambs (219 d) compared with shorter-fed lambs (93 d).

Direct comparison of results from previous lamb flavor literature and the present study is somewhat difficult because, in previous lamb flavor research, mutton flavor is commonly referred to as an off-flavor. Therefore, lamb flavor intensity was assessed, analyzed, and described as a separate variable, different from mutton and other specific off-flavor descriptors in previous lamb flavor research. The authors acknowledge the ideology of Field et al. (1983) that mutton flavor is a more intense, species-specific/lamb flavor commonly found in older sheep. Geographical region, and its influence on consumer taste profiles, may also be responsible for differences in lamb flavor results in the literature as consumer preferences for different lamb flavor intensity exist and have been reported to vary by geographical region (Prescott et al., 2001; Font i Furnols et al., 2006). Much of the reported flavor intensity research utilizes trained sensory panelists as a means to quantify differences among treatment arrangements on a standardized platform, but this approach also does not identify a consumer’s desired perception of a flavor profile. In the present study, a lamb-eating consumer panel approach was used to gain direct feedback on flavor and off-flavor intensity across the treatments. As viewed on the 0 to 100 scale by consumers, mean levels of flavor intensity were located toward the mild/medium-anchored end (30–40 units) of the measurement scale, an indication that feedlot-fed sheep did not produce meat products that were viewed as exhibiting a very-intense flavor profile across the studies. Results from the present study do not define a specific desired flavor profile but do demonstrate that management decisions can influence the flavor intensity of sheep meat due to the diets offered and harvest age of sheep. Providing this knowledge to the American lamb industry and disseminating the information to producers may allow the sheep industry to better distribute sheep meat products based on management decisions (diet consumed and the sheep’s age), thereby providing an opportunity to better meet the consumer’s expectation of a desirable lamb flavor intensity.

Currently, the American lamb industry uses a maturity-based carcass classification system that relies on break joint status. The break joints status of long-fed lambs in trial 2 indicated lamb maturity, as did chronological age based on known birth date records (Jaborek et al., 2018b). However, even with lamb maturity classification, long-fed lambs in trial 2 produced meat with a greater lamb flavor intensity and a greater off-flavor intensity. A classification system based on quality, which the consumer considers lamb flavor or taste as the most important attribute, is needed in the American lamb industry in order to consistently provide consumers with lamb products of a distinct lamb flavor intensity, such as mild, medium, and intense. Watkins et al. (2010) have previously measured the concentration of the branched-chain fatty acids deemed responsible for mutton or sheep-specific flavors and found that the concentration of these compounds increased with animal age, as did the lamb flavor intensity of ewes in trial 1 and long-fed lambs in trial 2 of the present study. In addition to sheep age, dietary management can affect lamb flavor intensity and the concentration of species-specific compounds (Watkins et al., 2010) as lamb flavor was greater for sheep consuming AP relative to WSC in the present study. Lastly, lamb flavor and off-flavors between ewe and wether lambs do not appear to be noticeably different for consumers. Watkins et al. (2014) reported that Australian consumers identified a greater overall liking of lamb when the concentrations of species-specific branched-chain fatty acids were at lower levels. Therefore, if an efficient and systematic technology for measuring the concentration of these species-specific compounds from carcasses in the packing plant were implemented, the American lamb industry would be able to provide American consumers with lamb products containing the lamb flavor profile they desire.

## CONCLUSION

Results from two trials reported in present study indicate that lamb flavor and off-flavor intensity of sheep meat can be affected through nutrition and by sheep age at harvest. Offering a forage-based diet of AP increased consumer perceptions of the lamb flavor and off-flavor intensity when compared with a whole-shelled corn-based diet. Lamb flavor intensity and off-flavor intensity increased as the age of sheep at harvest increased. Therefore, packers, producers, and lamb feeders in the American lamb industry can better meet the desired lamb flavor intensity sought by the consumer by managing the diet and age of sheep at harvest.
